# Exploring the complex relationship between vitamin K, gut microbiota, and warfarin variability in cardiac surgery patients

**DOI:** 10.1097/JS9.0000000000000673

**Published:** 2023-08-17

**Authors:** Ling Xue, Rajeev K. Singla, Qiong Qin, Yinglong Ding, Linsheng Liu, Xiaoliang Ding, Wenhao Qu, Chenrong Huang, Zhenya Shen, Bairong Shen, Liyan Miao

**Affiliations:** a Department of Pharmacy; b Department of Cardiovascular Surgery, The First Affiliated Hospital of Soochow University; c College of Pharmaceutical Sciences; d Institute for Interdisciplinary Drug Research and Translational Sciences, Soochow University, Suzhou; e National Clinical Research Center for Hematologic Diseases, The First Affiliated Hospital of Soochow University, Jiangsu; f Joint Laboratory of Artificial Intelligence for Critical Care Medicine, Department of Critical Care Medicine and Institutes for Systems Genetics, Frontiers Science Center for Disease-related Molecular Network, West China Hospital, Sichuan University, Chengdu, People’s Republic of China; g School of Pharmaceutical Sciences, Lovely Professional University, Phagwara, Punjab, India; h Department of Pharmacology, Faculty of Medicine, UPV/EHU, Spain

**Keywords:** drug microbiome interactions, gut microbiome, pharmacokinetics and pharmacodynamics, precision medicine, vitamin K, warfarin

## Abstract

**Background and objectives:**

Due to the high individual variability of anticoagulant warfarin, this study aimed to investigate the effects of vitamin K concentration and gut microbiota on individual variability of warfarin in 246 cardiac surgery patients.

**Methods:**

The pharmacokinetics and pharmacodynamics (PKPD) model predicted international normalized ratio (INR) and warfarin concentration. Serum and fecal samples were collected to detect warfarin and vitamin K [VK1 and menaquinone-4 (MK4)] concentrations and gut microbiota diversity, respectively. In addition, the patient’s medical records were reviewed for demographic characteristics, drug history, and CYP2C9, VKORC1, and CYP4F2 genotypes.

**Results:**

The PKPD model predicted ideal values of 62.7% for S-warfarin, 70.4% for R-warfarin, and 76.4% for INR. The normal VK1 level was 1.34±1.12 nmol/ml (95% CI: 0.33–4.08 nmol/ml), and the normal MK4 level was 0.22±0.18 nmol/ml (95% CI: 0.07–0.63 nmol/ml). The MK4 to total vitamin K ratio was 16.5±9.8% (95% CI: 4.3–41.5%). The S-warfarin concentration of producing 50% of maximum anticoagulation and the half-life of prothrombin complex activity tended to increase with vitamin K. Further, *Prevotella* and *Eubacterium* of gut microbiota identified as the main bacteria associated with individual variability of warfarin. The results suggest that an increase in vitamin K concentration can decrease anticoagulation, and gut microbiota may influence warfarin anticoagulation through vitamin K2 synthesis.

**Conclusion:**

This study highlights the importance of considering vitamin K concentration and gut microbiota when prescribing warfarin. The findings may have significant implications for the personalized use of warfarin. Further research is needed to understand better the role of vitamin K and gut microbiota in warfarin anticoagulation.

## Introduction

Highlights

- The fluctuations in vitamin K concentration in the body affect warfarin anticoagulant effects.
- The concentration of vitamin K1 and menaquinone K4 (one subtype of vitamin K2) and gut microbiota, which was the source of vitamin K2 was detected.
- Exploring the relationship between the concentration of vitamin K, gut microbiota, and the parameters of warfarin pharmacokinetics and pharmacodynamics

As a classical oral anticoagulant, warfarin is widely prescribed for preventing and treating thromboembolism in the clinic^1–3^. However, it has large individual variability and a narrow therapeutic index, and the daily dose variability may be up to 20-fold when achieving the target anticoagulant effect^4^. Inappropriate dosing significantly increases the risk of thromboembolism, bleeding, and hospitalization. Warfarin requires intensive monitoring of the blood clotting index (international normalized ratio, INR) to reduce these risks^5^. The narrow therapeutic index of warfarin makes it difficult to keep patients in an acceptable INR range for a long time^6^. Earlier research has shown several factors cause variability in warfarin, and numerous formulas have been created to optimize its dosage^7,8^. However, the most reasonable model should be based on the theory of pharmacokinetics and pharmacodynamics (PKPD) among these algorithms^9,10^. It should also be noted that some unknown factors were not included in those algorithms.

**Figure 1:**
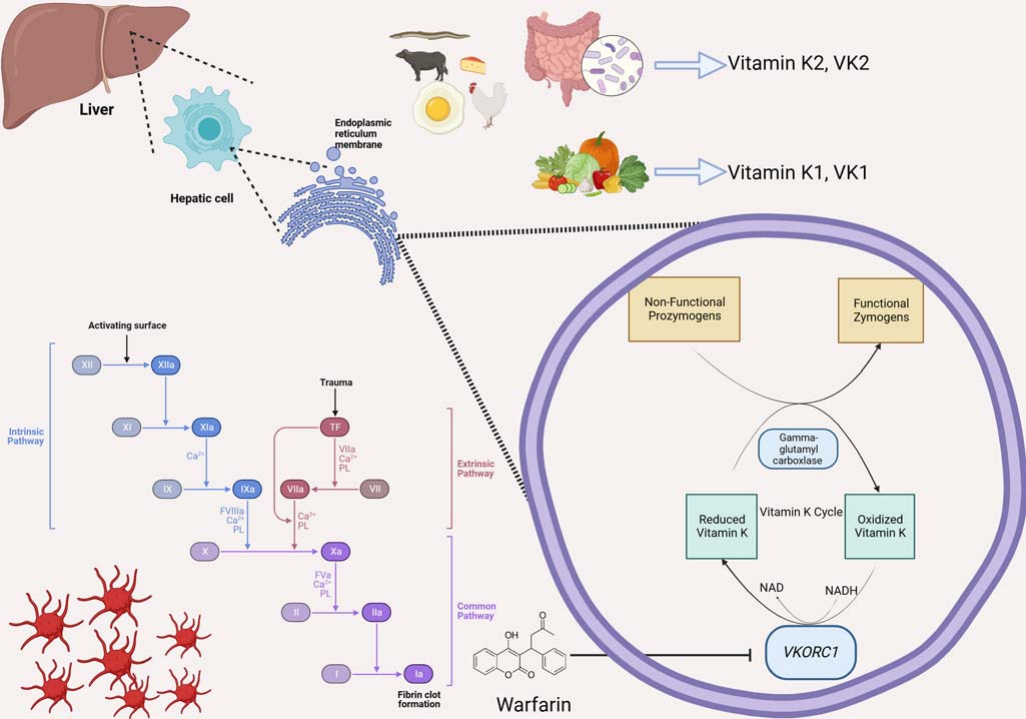
Role of vitamin K in activation of clotting factors and its modulation by warfarin. NAD, nicotinamide adenine dinucleotide; NADH, nicotinamide adenine dinucleotide (reduced form); VKORC1, vitamin K epoxide reductase complex.

Warfarin achieves its anticoagulant effect by disrupting the production of vitamin K-dependent clotting factors. It does this by inhibiting a key enzyme known as vitamin K epoxide reductase complex 1 (VKORC1)^11^. VKORC1 is a crucial enzyme in the vitamin K recycling process^12,13^. The cycle of vitamin K is mainly the oxidation and reduction process of naphthoquinone^14^. Vitamin K includes two primary forms: phylloquinone (vitamin K1, VK1) and menaquinones (vitamin K2, VK2). VK1 is a single compound with four isoprenoid residue side chains. However, VK2 has side chains with varying lengths between four and thirteen isoprene residues^15^. VK1 is exclusively found in plants, while VK2 (menaquinone-5 – menaquinone-13, MK5–MK13) is produced by a series of congeners synthesized by gram-positive bacteria in the human gastrointestinal tract^15,16^. Menaquinone-4 (MK4) is endogenously synthesized from VK1 in mammals^17^ and is found in animal products (Fig. 1).

All subtypes of vitamin K share a crucial functionality: acting as a co-factor for the post-translational enzyme γ-glutamate carboxylase. This universal role is predominantly attributed to the naphthoquinone structural framework they possess. γ-glutamate carboxylase is necessary to generate active coagulation factors II, VII, IX, and X^15,16^. The liver, known as the production site for vitamin K-dependent coagulation factors, was initially believed to be the main storage area for vitamin K^17^. Therefore, fluctuations in vitamin K concentration in the body may affect warfarin anticoagulant effects. However, information on the physiological and pharmacological roles of vitamin K *in vivo* is still limited. A challenge in optimizing therapy stems from the difficulty in detecting and monitoring vitamin K homologs in plasma and organs. This is due to their relatively low concentrations and the presence of numerous impurities, despite the critical importance of measuring vitamin K plasma concentrations^18^. Although the detection of vitamin K1 has been developed in plasma, detecting vitamin K2 is difficult in plasma. The human gut is considered a menaquinone reservoir and is estimated to contribute 10–50% of the vitamin K requirement in humans^15,19^. The situation of VK2 *in vivo* could be reflected by analyzing the gut microbiota composition. The gut microbiota is characterized by interindividual variability due to genetic and environmental factors^20^. In a previous study, we found that gut microbiota composition significantly changed post-antibiotics treatment in cardiac surgery patients^21^.

Furthermore, research has shown that antibiotics can influence both the diversity and abundance of gut microbiota. In rats that received antibiotic treatments, there was a decrease in the expression of certain liver metabolism enzymes, specifically CYP1A2, CYP2C9, and CYP3A4, as well as a reduction in P-glycoprotein (P-gp) expression^22^. INR increased in patients receiving warfarin combined with antibiotics (e.g. levofloxacin, gatifloxacin, trimethoprim–sulfamethoxazole) treatment^23,24^. Few studies explored the relationship between vitamin K concentration, gut microbiota, and warfarin. Reported studies mainly compared the vitamin K concentration with the warfarin sensitivity index^25–27^. The relationship between gut microbiota and warfarin was based on a multiple linear regression algorithm in our previous study^28^.

The PKPD model included the genotypes of CYP2C9, VKORC1, and CYP4F2, the combination drug of amiodarone, fluconazole, and vitamin K1, sex, age, body size, and type of heart valve, had been established in our previous study^10^. This study investigates the impact of vitamin K concentration and gut microbiota on the individual variability of warfarin, utilizing the PKPD model in patients undergoing cardiac surgery.

## Methods

### Patients and sampling

The study was approved by our organization’s Health Authority Ethics Committee, and we have followed the Declaration of Helsinki. All patients have given written informed consent. Patients underwent cardiac surgery for various reasons, including valvular heart disease, rheumatic valve disease, infectious endocarditis, aortic dissection (Stanford A or B), ascending aortic aneurysm, and congenital heart disease.

Patients need to be prescribed warfarin to prevent thromboembolism post-surgery. INR should be measured twice a week after warfarin administration in the hospital and once a month at least after the patient’s discharge from the hospital. The hospital clinical laboratory performed routine detection of the INR. Approximately 2 ml of blood was drawn in a tube with sodium citrate anticoagulation for INR detection. Firstly, prothrombin time (PT) detection was performed with the kit of STA-Neoplastine CI Plus or Thromborel S on an automated coagulation analyzer (STA-R (Evolution) or Sysmex CS-5100). Then, INR was derived by calculating the international sensitivity index (ISI) power of the patient’s PT to standard PT ratio. The ISI was recorded in the instructions of each batch kit. INR was also provided by the automated coagulation analyzer. Simultaneously, another ~2 ml of blood was drawn in a coagulation tube. These blood samples were centrifuged, and serum samples were stored at −80°C. The serum samples were utilized in further experiments to detect S-warfarin, R-warfarin, and vitamin K (VK1 and MK4) concentrations. In addition, CYP2C9 and VKORC1 were performed as part of standard clinical care. CYP4F2 was also genotyped for research purposes by multiplex PCR and sequencing.

Fecal samples of patients were also collected. Each patient was required to provide three fecal samples before antibiotics administration, antibiotics administration over 7 days, and antibiotics withdrawal over 7 days. All fecal samples were stored at −80°C until being used to analyze gut microbiota diversity.

### Bioanalysis

Total (bound plus unbound) serum concentrations of S-warfarin and R-warfarin were measured by liquid chromatography–mass spectrometry/mass spectrometry (LC–MS/MS) by a slight revision with the previously established detection method^10^ because the matrix was plasma in the previous detection method. Moreover, intraday and interday precision, recovery percent, and matrix effects were analyzed for change of sample matrix.

LC–MS/MS also measured the concentrations of VK1 and MK4. The detection method was validated from the linearity of calibration curves, lower limits of quantitation (LLOQ), intraassay and interassay precision, stability, recovery, and others, according to the Chinese biological sample analysis guideline in Chinese Pharmacopoeia. Due to the endogenous nature of vitamin K, 50% methanol and serum were used as the matrix of calibration curves and quality control and were compared with the standard substances, that is, deuterium-labeled VK1 (VK1-D7, Toronto Research Chemicals, Canada) and deuterium-labeled MK4 (MK4-D7, Toronto Research Chemicals, Canada). The process of serum sample preparation included liquid–liquid extraction by organic solvent and solid phase extraction by HyperSep SI (Thermo Scientific) as follows: (1) 600 µl ethanol and 10 µl internal standard (IS), which contained 20 ng/ml VK1-D7 and MK4-D7, were added into 200 µl serum sample; vortex-mixed 3 min; added with 1000 µl hexane; vortex-mixed 3 min; centrifuged at 14 000 rpm for 5 min at 4°C; 800 µl supernatant was transferred into a new plastic tube and evaporated to dryness for 30 min at 45°C in Integrated Speedvac (Thermo Scientific), then 800 µl hexane was added into the tube and mixed for 3 min. (2) 600 μl ether and hexane mixture (volume ratio 1:1) was used to activate the column of HyperSep SI, and repeat two times; 600 μl hexane was used to elute the column five times, due to the flow rate of each column was different, in order to ensure that the solvent of the column did not run dry when processing multiple samples, a few corresponding solvents with fast flow rate column was added in this step; all the sample of liquid–liquid extraction was added into the column, then 600 μl hexane was used to elute the column two times. Then 600 μl ether and hexane mixture (volume ratio 3:97) was used to elute the sample into a sample receiver. 1600 μl of sample eluent was transferred into a new plastic tube and evaporated to dryness for 50 min at 45°C in Integrated Speedvac. Finally, 100 μl ethanol was added to the tube to redissolve the sample, then centrifuged at 14 000 rpm for 5 min at 4°C. Then the supernatant was transferred into a vial of LC–MS/MS to detect the concentrations of VK1 and MK4.

According to the manufacturer’s protocol, bacterial genomic DNA was extracted from fecal samples using a genomic DNA purification kit (Omega Biotek, USA). We have amplified the V3–V4 hypervariable region of the bacterial 16S rRNA gene using PCR. The PCR product was extracted from a 2% agarose gel, purified using the AxyPrep DNA Gel Extraction Kit (Axygen Biosciences, USA), and quantified using a Quantus Fluorometer (Promega, USA). Purified PCR product amplicons were pooled in equimolar amounts and paired-end sequenced on MiSeq PE300 (Illumina, USA).

### Data analyzing

All the covariates included in the previously established PKPD model^10^ were screened from the present study. Then the predicted INR, S-warfarin, and R-warfarin concentrations were simulated by setting MAXEVAL=0 in NONMEM (version 7.5.0; ICON Development Solutions).

The predicted errors (PE) of INR and warfarin concentration were used to compare the bias of observed and predicted values by the previously established PKPD model^10^. The PE was calculated by Equation 1. For this study, a prediction was considered high if the PE was less than −20%, while an ideal prediction was from −20 to 20%. Conversely, a low prediction was defined as a PE more outstanding than 20%. Then, the contributions of vitamin K and gut microbiota to individual variability of warfarin were analyzed according to the PE.


1 $$\mathrm{PE}=\frac{\mathrm{Observed value}-\mathrm{Predicted value}}{\mathrm{Observed value}}\times 100\%.$$

The synthesis of prothrombin complex activity (PCA) was determined by factors II, VII, IX, and X. To describe the effects of S-warfarin and R-warfarin on PCA synthesis, a PD model based on the sigmoid *E*_max_ model was used, which assumed R-warfarin acted as a competitive antagonist of S-warfarin (Equation 2).


2 $$\mathrm{PD}=\frac{E_{\max}}{1+{\left(\frac{C_{S-\mathrm{warfarin}}}{{C50}_{\mathrm{\_S}-\mathrm{warfarin}}\times \left(1+\frac{C_{R-\mathrm{warfarin}}}{{\mathrm{RIC}50}_{\mathrm{\_R}-\mathrm{warfarin}}}\right)}\right)}^{-\mathrm{Hill}}}$$

where C50__S-warfarin_ was the S-warfarin concentration (*C*__S-warfarin_) of producing 50% of *E*_max_; RIC50__R-warfarin_ was the R-warfarin concentration (*C*__R-warfarin_) of producing a 50% increase in apparent C50__S-warfarin_.

The C50__S-warfarin_, half-life of PCA (T2PCA) and PD were derived from the PKPD model. Next, we analyzed the correlation between C50_S-warfarin, T2PCA, PD, and PE of INR with the total vitamin K concentration. The latter was computed as the combined mole concentration of VK1 and MK4.

The gut microbiota analysis was only performed according to the group of INR prediction bias, which was divided into high prediction, ideal prediction, and low prediction groups. The gut microbiota diversity was analyzed from *Alpha* diversity on the operational taxonomic unit level among the three groups. The parameters of *Alpha* diversity were tested by following *Mann–Whitney U* on GraphPad (version 8.0). The *Kruskal–Wallis H* test was used to compare bacterial genera across three groups. Partial least squares discriminant analysis (PLS-DA) was used to assess differences between groups at the operational taxonomic unit level. All data were analyzed using the Majorbio Cloud Platform, a free online platform (http://www.majorbio.com).

## Results

### Patient characteristics

A total of 246 patients, with 1469 observed warfarin concentrations, 3018 observed INRs, and 622 fecal samples, were enrolled in the present study. In addition, the demographic characteristics of the patients, indication for surgery, and drug history were recorded from the patient’s medical records (Table 1).

**Table 1:** Demographic characteristics of patients.

Items	Value
Age (year)	58±13
Height (cm)	164.7±8.8
Weight (kg)	63.9±11.8
Sex	
Male	157 (63.8%)
Female	89 (36.2%)
Race	
Han	244 (99.2%)
Non-Han	2 (0.8%)
CYP2C9 (rs1057910)	
*1/*1	227 (92.3%)
*1/*3	19 (7.7%)
VKORC1 (rs9923231)	
GG	3 (1.2%)
GA	33 (13.4%)
AA	210 (85.4%)
CYP4F2 (rs2108622)	
CC	147 (59.8%)
CT	82 (33.3%)
TT	17 (6.9%)
Type of surgery	
DVR	25 (10.2%)
AVR	36 (14.6%)
MVR	97 (39.4%)
TVR	3 (1.2%)
Bentall	31 (12.6%)
Wheat	17 (6.9%)
Valve plasty	8 (3.3%)
TEVAR	16 (6.5%)
AVARMB	13 (5.3%)
Type of valve	
Mechanical valve prosthesis	86 (35.0%)
Bioprosthetic heart valves	123 (50.0%)
No valve replacement	37 (15.0%)

AVARMB, artificial vascular replacement of ascending aorta and modified branching; AVR, aortic valve replacement; DVR, aortic and mitral valve replacement; MVR, mitral valve replacement; TEVAR, thoracic endovascular aortic repair; TVR, tricuspid valve replacement; Valve plasty, mitral or/and tricuspid valve plasty.

### Bioanalysis

The linear range of S-warfarin and R-warfarin concentrations detection was revised to 25–2000 ng/ml, and the three levels of quality control concentrations (QCs) were set as 75, 500, and 1600 ng/ml. The intraday and interday precision, as indicated by the coefficient of variation (CV), was between 3.3 and 8.7%, and the assay accuracy was between 87.4 and 93.7% for the three levels of QC of S-warfarin and R-warfarin. The recovery percent at 75, 500, and 1600 ng/ml concentrations was 56.7, 63.1, and 63.7% for S-warfarin and 55.0, 61.7, and 63.5% for R-warfarin, respectively. The matrix effects at 75, 500, and 1600 ng/ml concentrations were 97.7, 93.4, and 91.4% for S-warfarin and 96.0, 93.5, and 92.4% for R-warfarin, respectively (Supplementary Table S1, Supplemental Digital Contents 1, http://links.lww.com/JS9/A915 and Supplementary Table S2, Supplemental Digital Contents 2, http://links.lww.com/JS9/A916).

VK1 and MK4 concentrations detection linear range were 0.05–5.0 ng/ml. Six samples were prepared for each concentration level during the detection method validation. The result of the detection method could be accepted when the determined concentration accuracy of two-thirds of the samples was over 80% for LLOQ or 85% for the three concentration levels of 0.15, 0.5, and 4.0 ng/ml. As vitamin K is an endogenous substance, obtaining a blank serum matrix without any vitamin K proved challenging. Therefore, VK1-D7 and MK4-D7 were added to 50% methanol and blank serum as standard substances to compare the differences in the matrix. The results showed that 50% methanol and serum, as the matrix of calibration curves and QC samples, had no difference (Supplementary Table S3, Supplemental Digital Content 3, http://links.lww.com/JS9/A917). Therefore, the results suggest that using 50% methanol and a blank serum matrix without vitamin K did not impact the detection of vitamin K.

Moreover, the sample processing for calibration curves and QC samples required only one step of liquid–liquid extraction, which saved time compared to the time-consuming solid-phase extraction method. Therefore, 50% methanol was chosen as the matrix of calibration curves and QC samples. The intraday and interday precision and stability results were shown in Supplementary Table S4 (Supplemental Digital Content 4, http://links.lww.com/JS9/A918) and Supplementary Table S5 (Supplemental Digital Content 5, http://links.lww.com/JS9/A919) for detection method validation. The CV of intraday and interday precision was between 3.5 and 14.9% for VK1 and between 3.8 and 11.1% for MK4. The assay accuracy was between 100.2 and 114.7% for VK1 and 94.9 and 102.2% for MK4.

### Predicted error by PKPD model

**Figure 2:**
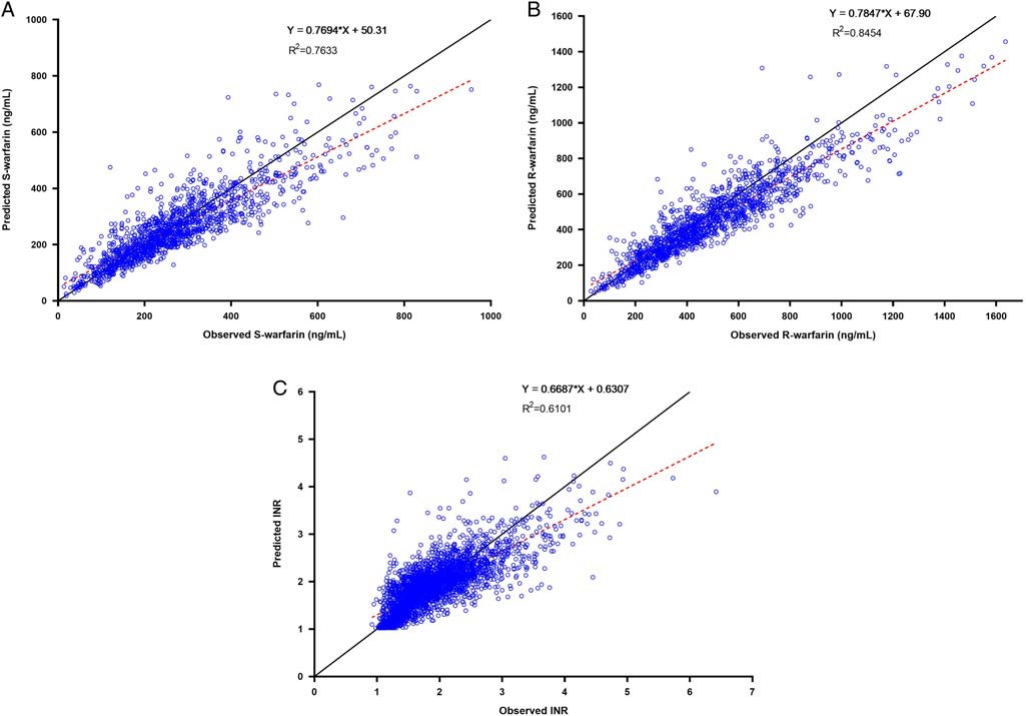
Scatter plot of observed value versus predicted value by PKPD (pharmacokinetics and pharmacodynamics) model. (A) S-warfarin concentration; (B) R-warfarin concentration; (C) international normalized ratio.

The results of INR and warfarin concentration predicted bias by the PKPD model are shown in Table 2 and Figure 2. Thirteen patients were administered voriconazole in the present study. Voriconazole was not included as a covariate in the previously established PKPD model because no patients were administered voriconazole in the previous study. In order to decrease the predicted bias of the model, voriconazole was considered as fluconazole while introducing the covariate of fluconazole into the PKPD model because voriconazole also inhibits liver enzymes. Interoccasion variability (IOV) of the volume of distribution S-warfarin (*V*__S-warfarin_) and bioavailability (*F*) were omitted when prediction values of S-warfarin and R-warfarin concentrations and INR were derived from the PKPD model. The percent of ideal prediction was 62.7, 70.4, and 76.4% for S-warfarin, R-warfarin concentrations, and INR, respectively. There were still 15.3% predictions on the high side and 8.3% on the low side for INR.

**Table 2:** PE of INR and warfarin concentration by the PKPD model.

	High prediction	Ideal prediction	Low prediction
S-warfarin (*n*=1469)	250 (17.0%)	921 (62.7%)	298 (20.3%)
R-warfarin (*n*=1469)	139 (9.5%)	1035 (70.4%)	295 (20.1%)
INR (*n*=3018)	462 (15.3%)	2305 (76.4%)	251(8.3%)

INR, international normalized ratio; PE, predicted errors; PKPD, pharmacokinetics and pharmacodynamics.

### Effect of vitamin K concentration on warfarin

The datasets collected before warfarin administration and after vitamin K1 were excluded as a concomitant drug due to high INR for vitamin K concentration detection. One thousand four hundred twenty (1420) vitamin K concentrations were used to explore the relationship with warfarin. The normal level was 1.34±1.12 nmol/ml (95% confidence interval (CI): 0.33–4.08 nmol/ml) for VK1 and 0.22±0.18 nmol/ml (95% CI: 0.07–0.63 nmol/ml) for MK4 *in vivo* of these patients. The MK4 to total vitamin K ratio was 16.5±9.8% (95% CI: 4.3–41.5%).

**Figure 3:**
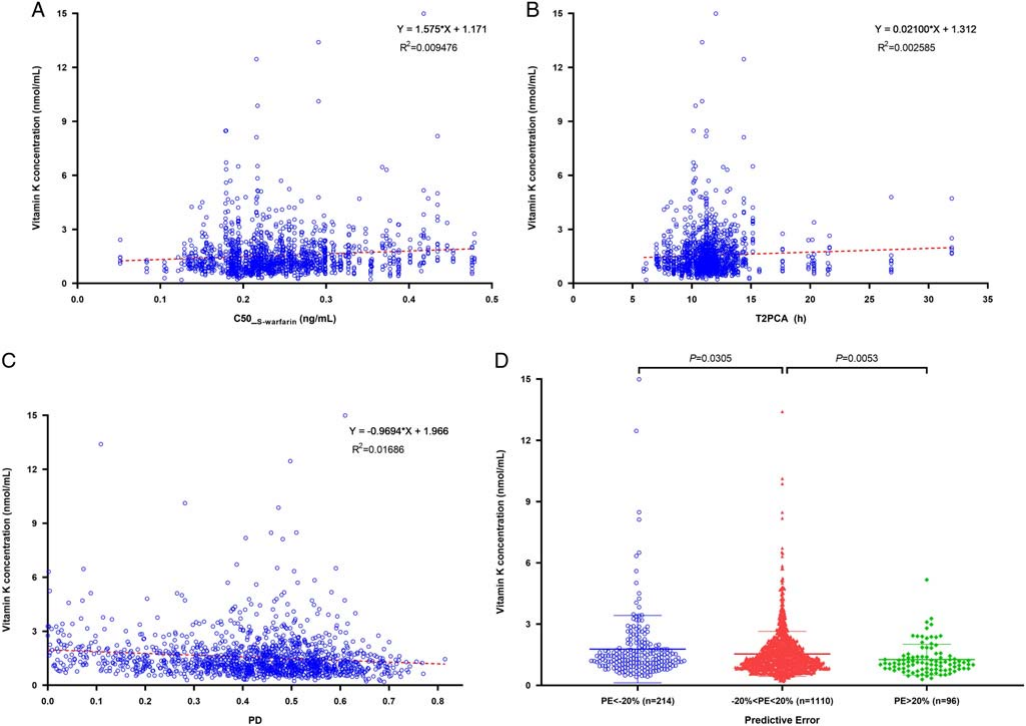
The relationship between vitamin K concentration and the parameters of warfarin PKPD (pharmacokinetics and pharmacodynamics).

Since vitamin K circulation *in vivo* was involved in the clotting process, the pharmacodynamics parameters associated with warfarin for C50__S-warfarin_, T2PCA, PD, and PE of INR were analyzed to explore the effect of the total vitamin K concentration (sum of VK1 and MK4 mole concentration). The parameters of C50__S-warfarin_, T2PCA, and PD were estimated as 0.24±0.07 mg/l (95% CI: 0.13–0.43), 11.6±2.9 h (95% CI: 7.6–20.3), 0.42±0.16 (95% CI: 0.04–0.69) using the PKPD model, respectively. The results of the relationship between the total vitamin K concentration and C50__S-warfarin_, T2PCA, PD, and PE of INR are shown in Figure 3. C50__S-warfarin_ and T2PCA tended to increase, while PD tended to decrease with an increasing total vitamin K concentration. The total vitamin K concentration in the high prediction group (*n*=214, mean±SD: 1.77±1.65 nmol/ml, 95% CI:0.49–6.44) was significantly higher than that in the ideal prediction group (*n*=1110, mean±SD: 1.54±1.10 nmol/ml, 95% CI: 0.47–4.48, *P*=0.0305), while the total vitamin K concentration in the low prediction group (*n*=96, mean±SD: 1.26±0.75 nmol/ml, 95% CI: 0.39–3.20) was significantly lower than that in the ideal prediction group (*P*=0.0053).

### Effect of gut microbiota on warfarin

The effect of gut microbiota on the pharmacodynamics of warfarin was analyzed by comparing gut microbiota and INR prediction deviation in the present study. A total of 622 fecal samples were collected in this study, of which 176 were collected pre-warfarin administration, and the remaining 446 were taken post-warfarin administration. Only the fecal samples of post-warfarin administration were analyzed to explore the relationship between gut microbiota and warfarin. 61, 330, and 55 samples were in high, ideal, and low predictions for INRs’ PE, respectively.

**Figure 4:**
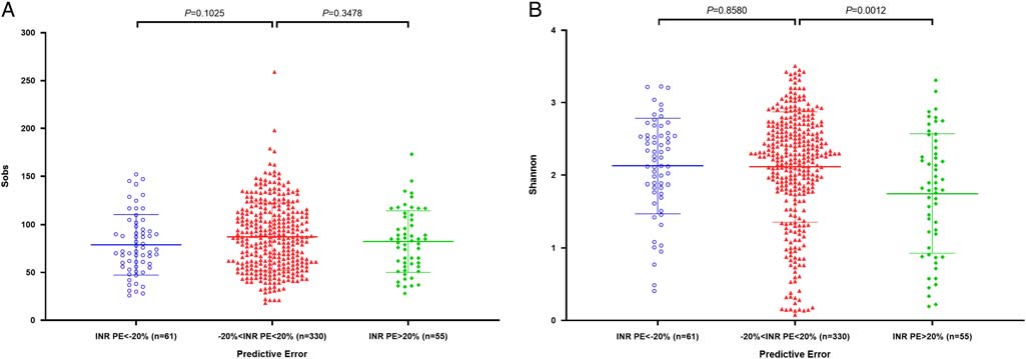
The community richness and diversity of each group of gut microbiota. (A) Sobs index; (B) Shannon index.

**Figure 5:**
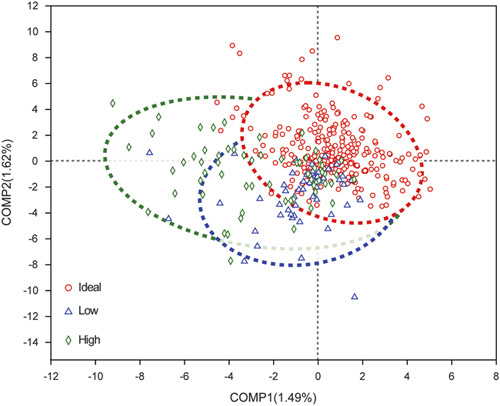
Partial least squares discriminant analysis (PLS-DA) of gut microbiota in each group. The dotted line represents the 95% confidence interval line.

**Figure 6:**
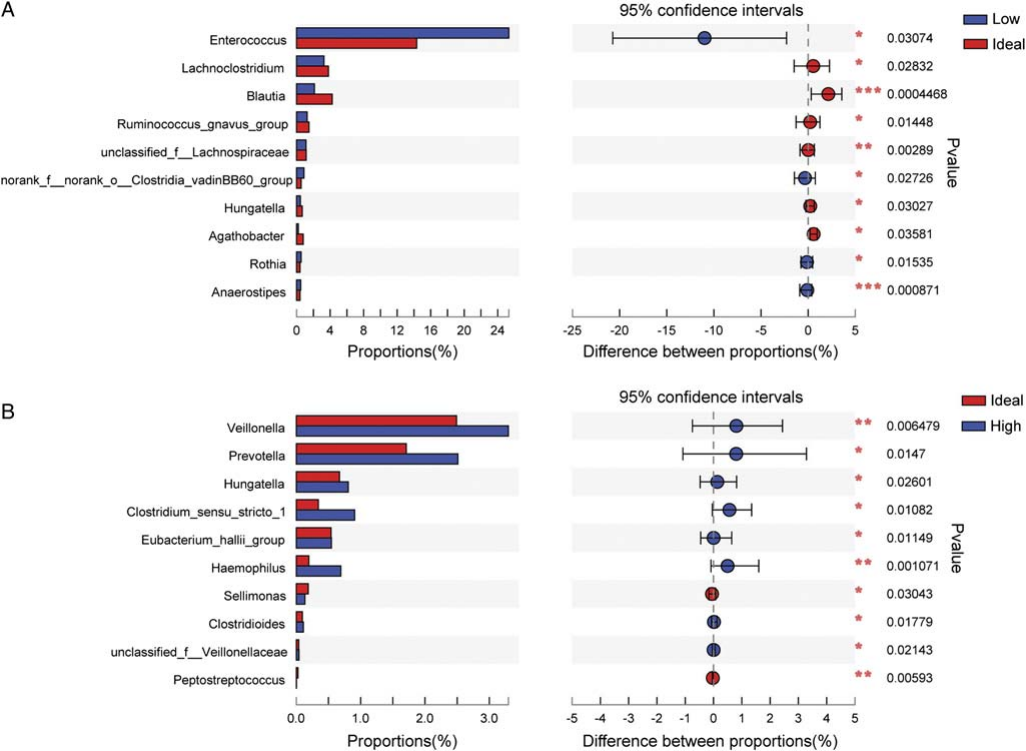
The top 10 bacterial species with statistical significance in each group. (A) Low prediction versus ideal prediction; (B) Ideal prediction versus high prediction. *P<0.05, **P<0.01, ***P<0.001.

The results of the community richness and diversity for gut microbiota were shown in Figure 4 according to INRs’ PE grouping. The community richness of gut microbiota, as indicated by the index of *Sobs*, had no difference among the three groups (high prediction group: mean±SD: 78.9±31.6, 95% CI: 27.1–149.3; ideal prediction group: mean±SD: 87.2±35.3, 95% CI: 31.3–153.7; low prediction group: mean±SD: 82.3±32.1, 95% CI: 30.8–161.8). The community diversity of gut microbiota, as indicated by the index of *Shannon*, had no difference between the high (mean±SD: 2.13±0.66, 95% CI: 0.45–3.22) and ideal prediction groups (mean±SD: 2.11±0.76, 95% CI: 0.16–3.34) (Fig. 4A, *P*>0.05). However, the *Shannon* of the low prediction group (mean±SD: 1.75±0.82, 95% CI: 0.20–3.25) was significantly lower than the ideal prediction group (Fig. 4B, *P*=0.0012). The result of PLS-DA showed that the three groups had some differences (Fig. 5). The top 10 bacterial species with significant differences between every two groups for INRs’ PE are shown in Figure 6. The results showed that compared with the ideal group, the relative abundance of *Lachnoclostridium*, *Blautia*, *Ruminococcus_gnavus*, *unclassified_f_Lachnospiraceae*, *Hungatella*, and *Agathobacter* significantly decreased. However, the relative abundance of *Enterococcus*, *norank_f_norank_o_Clostridia_vadinBB60*, *Rothia*, and *Anaerostipes* significantly increased in the low group (Fig. 6A, *P*<0.05). Furthermore, compared with the ideal group, the relative abundance of *Veillonella*, *Prevotella*, *Hungatella*, *Clostridium_sensu_stricto_1*, *Eubacterium_hallii*, *Haemophilus*, *Clostridioides*, and *unclassified_f_Veillonellaceae* significantly increased. However, the relative abundance of *Sellimonas* and *Peptostreptococcus* significantly decreased in the high group (Fig. 6B, *P*<0.05).

## Discussion

Warfarin, a coumarin anticoagulant, has been widely used in clinical practice for several decades. Various dosing algorithms have been established based on demographic, clinical, and environmental factors, with many relying on multivariate linear regression (MLR). The median coefficient of determination (*R* ^2^), which reflects the proportion of total interindividual variability in warfarin dose requirements, was found to be 43% (range 2–96%) for the MLR model^29^. PKPD model had better predictive performance than other algorithms. This study explored the influence of vitamin K concentration and gut microbiota on individual variability of warfarin based on the previously established PKPD model^10^. As expected, vitamin K concentration and gut microbiota had been indicated that be associated with warfarin anticoagulation.

The anticoagulation of warfarin interferes with the circulation of vitamin K^12,13^. The source of vitamin K includes diet (VK1 and MK4) and gut microbiota (MK5–MK13)^16,30^. When VK1 was administrated as a concomitant drug against high INR by intravenous drip, the concentration of VK1 significantly increased. Some serum samples were collected at about 2 h and some samples on the second morning after the end of the VK1 intravenous drip. The results of VK1 concentrations were very high for about 2 h after the end of the VK1 intravenous drip (*n*=7; mean±SD: 299.68±209.15 nmol/ml; 95% CI: 94.68–589.03 nmol/ml). VK1 concentrations significantly dropped for the samples collected on the second morning (*n*=15; mean±SD: 6.23±4.14 nmol/ml; 95% CI: 2.79–15.79 nmol/ml). However, the normal level of VK1 concentrations was 1.34±1.12 nmol/ml (95% CI: 0.33–4.08 nmol/ml) for those patients without concomitant VK1. The VK concentrations of these samples were not included in the analysis because VK1 was used as a covariate in the PKPD model. It was assumed that the effect of VK1 on warfarin anticoagulant lasted until the next day^10^. The present study mainly explored the relationship between normal vitamin K levels and warfarin anticoagulant effect in patients. MK4 accounted for 16.5% of the total vitamin K in the body. These results are consistent with previous reports^15^. Following the administration of warfarin, a significant reduction in vitamin K concentration was observed (mean±SD: from 2.47±1.90 to 1.56±1.19 nmol/ml, *P*<0.05). This may be attributed to an increased usage of vitamin K within the body post-warfarin administration.

The dataset for the present study was slightly different from previous studies. Voriconazole was not included as a covariate in the previously established PKPD model because no patients were administered voriconazole in the previous study. However, in the present study, some patients were treated with voriconazole for fungal infections. Voriconazole has an inhibition for specific CYP450 isoenzymes^31^, and concomitant administration of voriconazole showed that it potentiated warfarin anticoagulation^32^. To reduce the prediction error of the pharmacokinetic–pharmacodynamic (PKPD) model, it was hypothesized that voriconazole has a similar impact on warfarin’s anticoagulation as fluconazole. Additionally, it was assumed in this study that the inhibitory effect of voriconazole on warfarin’s pharmacokinetics persists for a week. Although the IOV of the volume of distribution S-warfarin (*V*__S-warfarin_) and bioavailability (*F*) was estimated in the development process of the PKPD model, this portion of unexplained variability was demonstrated that could not increase the accuracy of model predictions when calculating the forecast dose^33^. Therefore, the IOV parameters of *V*__S-warfarin_ and *F* were set to 0 when predicting the warfarin concentration and INR using the PKPD model.

The vitamin K cycle *in vivo* consists of three steps: (1) the reduction of vitamin K quinone to vitamin K hydroquinone (VKH_2_); (2) the oxidation of VKH_2_ to vitamin K epoxide (VKO); (3) the reduction of VKO to vitamin K quinone. The second step was associated with forming activated clotting factors, and the first and third steps were sensitive to warfarin^13,34^. Furthermore, the AIFM2 gene encodes the ferroptosis suppressor protein 1 (FSP1), which plays a role in the initial stage of the vitamin K cycle and maintains coagulation function in the presence of anticoagulant drugs^34^. Therefore, increased dietary vitamin K intake could increase the first and second steps of the vitamin K cycle in patients with warfarin administration. As a result, active clotting factor formation increases, leading to decreased INR in patients. In clinical practice, we often found that INR significantly decreased when some patients had eaten some vegetables with vitamin K-rich, such as cabbage, lettuce, and broccoli^15,35,36^, during the treatment of warfarin. Patients were told to keep their eating habits as stable as possible while taking warfarin and not to suddenly eat vitamin K-rich foods, which may cause fluctuations in vitamin K concentration in the body. After adjusting the patient’s dietary habits, the INR was tested again to return to the target range without adjusting the warfarin dose. This phenomenon could be explained as the change in the pharmacodynamic parameters of warfarin by vitamin K. The present study showed that more warfarin was required to achieve the same anticoagulant effect due to C50__S-warfarin_ increase, the half-life of the PCA lengthened, and the predicted INR was overestimation by the PKPD model as the increase of vitamin K concentration.

As mentioned above, gut microbiota was also considered an essential source of vitamin K2 in addition to the diet. VK2 is a complex compound comprising a group of menaquinone homologs^37^. The level of VK2 was indirectly reflected by analyzing the composition of gut microbiota. The *Prevotella* and *Bacteroides* were the most influential taxa underlying menaquinotypes^19,38^. *Prevotella* was considered the bacterial genus for the biosynthesis of MK5–MK7 and MK11–MK13^19,38,39^. *Bacteroides* mainly biosynthesized large amounts of MK10–11 and minor amounts of MK7–MK9 and MK12^15,17^, while *Eubacterium* produces MK6^15^. Both *Escherichia* and *Klebsiella* are known to biosynthesize MK8^19^. In addition, *Bifidobacterium*, *Lactococcus lactis*, *Leuconostoc*, *Streptococcus* ^40^, *Bacillus cereus*, *Staphylococcus aureus* ^41^ also produce menaquinone. The present study showed that *Prevotella* and *Eubacterium_hallii* were higher in the high group for INRs’ PE. This may cause menaquinone content to increase, and actual INR observation is lower than prediction by the PKPD model. Our team members reported that the gut microbiota associated with warfarin anticoagulation consisted of *Enterococcus* and *Escherichia* ^28^. The results of two studies by our team showed that *Enterococcus* was higher in the low prediction group. *Enterococcus* is an important nosocomial pathogen, and the abundance of *Enterococcus* increase is associated with antibiotics^42^. However, *Escherichia* had no difference among the three groups of INRs’ PE, and the cause of this difference might be the different sample sizes and different grouping methods. The number was 80, and grouped according to the MLR model in the previous study^28^.

In addition to the gut microbiota’s role in warfarin anticoagulation through vitamin K2 biosynthesis, it can also impact warfarin pharmacokinetics. The oral administration of drugs faces multiple barriers, including physicochemical barriers, transporters, metabolizing enzymes, and bacteria, before reaching systemic circulation. Gut bacteria can directly metabolize various drugs before absorption. Moreover, the gut microbiome indirectly influences drug metabolism by regulating metabolizing enzyme activity. This regulation may occur by binding the pregnancy X receptor (PXR), a known transcriptional regulator of CYP3A in the liver, to changes in the gut microbiome^43^. Our team members conducted some experiments (data not reported) demonstrating that gut microbiota could not metabolize warfarin. However, exposure to S-warfarin was significantly increased in mice treated with antibiotics. The mechanism should be further investigated for the relationship between gut microbiota and warfarin pharmacokinetics.

The limitations were that the present study was merely a clinical data analysis, and the effect of vitamin K concentration and gut microbiota on the parameters of warfarin PKPD was only a qualitative analysis. The mechanisms of gut microbiota that influence warfarin anticoagulation should be further explored. Furthermore, the quantitative relationship between vitamin K concentration, gut microbiota, and warfarin anticoagulation should be established in the future.

In conclusion, this study investigated the impact of vitamin K concentration and gut microbiota on individual variability in warfarin response. As anticipated, higher vitamin K concentrations correlated with increased C50_S-warfarin and T2PCA levels, decreasing warfarin anticoagulation. In addition, the gut microbiota’s synthesis of vitamin K2, primarily through *Prevotella* and *Eubacterium*, influenced warfarin anticoagulation.

## Ethical approval

The study was approved by the Health Authority Ethics Committee of the First Affiliated Hospital of Soochow University (2019011).

## Consent

Written informed consent was obtained from the patient for the publication of this case report and accompanying images. A copy of the written consent is available for review by the Editor-in-Chief of this journal on request.

## Sources of funding

This work was supported by the National Natural Science Foundation of China (grant number 81803628), Jiangsu Provincial Medical Key Discipline (ZDXK202247), Key R&D Program of Jiangsu Province (BE2021644), Suzhou Health Leading Talent (GSWS2019001), the National Clinical Research Center for Hematologic Diseases, The First Affiliated Hospital of Soochow University (2020 WSC07), and the Priority Academic Program Development of the Jiangsu Higher Education Institutes (PAPD).

## Author contribution

L.X.: designing the study, data collection, data analysis and interpretation, and writing and revising the paper; R.K.S.: visualization, writing, and revising the paper; Q.Q. and Y.D.: data collection and data interpretation; L.L.: detecting the concentrations of vitamin K1 and menaquinones K4; X.D.: detecting the concentrations of S-warfarin and R-warfarin and genotyping; W.Q.: detecting the concentrations of S-warfarin, R-warfarin, vitamin K1, and menaquinones K4; C.H.: designing the study and data interpretation; Z.S., B.S., and L.M.: designing the study, data interpretation, and revising the paper.

## Conflicts of interest disclosure

The authors have no conflicts of interest relevant to this study.

## Research registration unique identifying number (UIN)

Exploring the complex relationship between vitamin K, gut microbiota, and warfarin variability in cardiac surgery patients (https://www.researchregistry.com/browse-the-registry#home, researchregistry9064).

## Guarantor

Liyan Miao, Department of Pharmacy, The First Affiliated Hospital of Soochow University, Suzhou, China; E-mail: miaolysuzhou@163.com.

## Data availability statement

All the critical information is already available in the manuscript and supplementary files. However, authors are still ready to share the raw data if the proper channel for the inquiry is followed, which will be routed through journal and affiliation authorities.

## Provenance and peer review

This paper was not invited.

## Supplementary Material

SUPPLEMENTARY MATERIAL
