# Supplementary material for: Exploring the complex relationship between vitamin K, gut microbiota, and warfarin variability in cardiac surgery patients
Source: Int J Surg. 2023 Aug 17;109(12):3861–71. doi: 10.1097/JS9.0000000000000673 (PMC10720796; doi:10.1097/JS9.0000000000000673)
Supplement: SUPPLEMENTARY MATERIAL [file js9-109-3861-s001.docx]

Table S1. The Intra- and interassay precision of warfarin concentration for the revised validation of detection method by LC-MS/MS

| Precision | Warfarin | Standard concentration (ng/mL) | Determined concentration  (Mean±SD, ng/mL) | CV | Accuracy |
| --- | --- | --- | --- | --- | --- |
| Intra-assay | S-warfarin | 75 | 65.5±3.05 | 4.7% | 87.4% |
|  |  | 500 | 464.3±33.3 | 7.2% | 92.9% |
|  |  | 1600 | 1474.3±48.5 | 3.3% | 92.1% |
|  | R-warfarin | 75 | 66.0±3.05 | 4.6% | 88.0% |
|  |  | 500 | 447.9±38.8 | 8.7% | 89.6% |
|  |  | 1600 | 1418.3±62.4 | 4.4% | 88.6% |
| Inter-assay | S-warfarin | 75 | 66.6±2.69 | 4.0% | 88.7% |
|  |  | 500 | 458.3±24.6 | 5.4% | 91.7% |
|  |  | 1600 | 1499.4±85.6 | 5.7% | 93.7% |
|  | R-warfarin | 75 | 66.8±3.10 | 4.6% | 89.0% |
|  |  | 500 | 458.0±27.1 | 5.9% | 91.6% |
|  |  | 1600 | 1476.3±93.6 | 6.3% | 92.3% |
