# Supplementary material for: Exploring the complex relationship between vitamin K, gut microbiota, and warfarin variability in cardiac surgery patients
Source: Int J Surg. 2023 Aug 17;109(12):3861–71. doi: 10.1097/JS9.0000000000000673 (PMC10720796; doi:10.1097/JS9.0000000000000673)
Supplement: SUPPLEMENTARY MATERIAL [file js9-109-3861-s002.docx]

Table S2. The recovery and matrix effects of warfarin concentration for the revised validation of detection method by LC-MS/MS

| Warfarin | Standard concentration (ng/mL) | Recovery | Matrix effects |
| --- | --- | --- | --- |
| S-warfarin | 75 | 56.7% | 97.7% |
|  | 500 | 63.1% | 93.4% |
|  | 1600 | 63.7% | 91.4% |
| R-warfarin | 75 | 55.0% | 96.0% |
|  | 500 | 61.7% | 93.5% |
|  | 1600 | 63.5% | 92.4% |
