# Supplementary material for: Exploring the complex relationship between vitamin K, gut microbiota, and warfarin variability in cardiac surgery patients
Source: Int J Surg. 2023 Aug 17;109(12):3861–71. doi: 10.1097/JS9.0000000000000673 (PMC10720796; doi:10.1097/JS9.0000000000000673)
Supplement: SUPPLEMENTARY MATERIAL [file js9-109-3861-s003.docx]

Table S3. The different matrices of vitamin K for the validation of the detection method by LC-MS/MS

|  | Different matrix | Standard concentration (ng/mL) | Determined concentration  (Mean±SD, ng/mL) | CV | Accuracy |
| --- | --- | --- | --- | --- | --- |
| VK1-D7 | 50% methanol | 0.05 | 0.0543±0.0015 | 2.7% | 108.7% |
|  |  | 0.15 | 0.158±0.0078 | 4.9% | 105.5% |
|  |  | 0.50 | 0.512±0.0241 | 4.7% | 102.5% |
|  |  | 4.00 | 4.09±0.136 | 3.3% | 102.2% |
|  | Serum | 0.05 | 0.0573±0.0057 | 10.0% | 114.7% |
|  |  | 0.15 | 0.165±0.0084 | 5.1% | 109.9% |
|  |  | 0.50 | 0.484±0.0226 | 4.7% | 96.7% |
|  |  | 4.00 | 3.86±0.337 | 8.7% | 96.4% |
| MK4-D7 | 50% methanol | 0.05 | 0.0447±0.005 | 11.3% | 89.4% |
|  |  | 0.15 | 0.142±0.0064 | 4.5% | 94.4% |
|  |  | 0.50 | 0.481±0.0389 | 8.1% | 96.2% |
|  |  | 4.00 | 4.03±0.354 | 8.8% | 100.7% |
|  | Serum | 0.05 | 0.0544±0.0056 | 10.4% | 108.8% |
|  |  | 0.15 | 0.173±0.0231 | 13.4% | 115.0% |
|  |  | 0.50 | 0.525±0.0299 | 5.7% | 105.0% |
|  |  | 4.00 | 4.14±0.318 | 7.7% | 103.4% |
