# Supplementary material for: Exploring the complex relationship between vitamin K, gut microbiota, and warfarin variability in cardiac surgery patients
Source: Int J Surg. 2023 Aug 17;109(12):3861–71. doi: 10.1097/JS9.0000000000000673 (PMC10720796; doi:10.1097/JS9.0000000000000673)
Supplement: SUPPLEMENTARY MATERIAL [file js9-109-3861-s004.docx]

Table S4. The Intra- and interassay precision of vitamin K for the validation of detection method by LC-MS/MS

| Precision | Vitamin K | Standard concentration (ng/mL) | Determined concentration  (Mean±SD, ng/mL) | CV | Accuracy |
| --- | --- | --- | --- | --- | --- |
| Intra-assay | VK1 | 0.05 | 0.0573±0.0086 | 14.9% | 114.7% |
|  |  | 0.15 | 0.154±0.0054 | 3.5% | 102.7% |
|  |  | 0.50 | 0.515±0.0359 | 7.0% | 103.1% |
|  |  | 4.00 | 4.01±0.143 | 3.6% | 100.2% |
|  | MK4 | 0.05 | 0.0481±0.0045 | 9.3% | 96.2% |
|  |  | 0.15 | 0.142±0.0054 | 3.8% | 94.9% |
|  |  | 0.50 | 0.48±0.0348 | 7.3% | 95.9% |
|  |  | 4.00 | 3.89±0.194 | 5.0% | 97.3% |
| Inter-assay | VK1 | 0.05 | 0.0534±0.00628 | 11.8% | 106.7% |
|  |  | 0.15 | 0.157±0.0143 | 9.1% | 104.8% |
|  |  | 0.50 | 0.523±0.0291 | 5.6% | 104.6% |
|  |  | 4.00 | 4.13±0.284 | 6.9% | 103.3% |
|  | MK4 | 0.05 | 0.0511±0.00569 | 11.1% | 102.2% |
|  |  | 0.15 | 0.147±0.0118 | 8.0% | 98.2% |
|  |  | 0.50 | 0.486±0.0367 | 7.5% | 97.3% |
|  |  | 4.00 | 3.83±0.258 | 6.7% | 95.9% |
