# Supplementary material for: Exploring the complex relationship between vitamin K, gut microbiota, and warfarin variability in cardiac surgery patients
Source: Int J Surg. 2023 Aug 17;109(12):3861–71. doi: 10.1097/JS9.0000000000000673 (PMC10720796; doi:10.1097/JS9.0000000000000673)
Supplement: SUPPLEMENTARY MATERIAL [file js9-109-3861-s005.docx]

Table S5. The stability of vitamin K for the validation of the detection method by LC-MS/MS

| Stability | Vitamin K | Standard concentration (ng/mL) | Determined concentration  (Mean±SD, ng/mL) | CV | Accuracy |
| --- | --- | --- | --- | --- | --- |
| After 24h placement at sampler | VK1 | 0.15 | 0.162±0.0157 | 9.7% | 108.1% |
|  |  | 4.00 | 4.39±0.314 | 7.1% | 109.9% |
|  | MK4 | 0.15 | 0.166±0.02 | 12.0% | 110.7% |
|  |  | 4.00 | 4.36±0.287 | 6.6% | 108.9% |
| Sample placement after 6h at room temperature | VK1 | 0.15 | 0.168±0.0154 | 9.1% | 112.3% |
|  |  | 4.00 | 4.37±0.172 | 3.9% | 109.2% |
|  | MK4 | 0.15 | 0.159±0.0155 | 9.8% | 105.8% |
|  |  | 4.00 | 4.14±0.265 | 6.4% | 103.4% |
| Sample placement after 3 days at 4℃ | VK1 | 0.15 | 0.158±0.0114 | 7.2% | 105.3% |
|  |  | 4.00 | 4.04±0.262 | 6.5% | 101.1% |
|  | MK4 | 0.15 | 0.149±0.0159 | 10.7% | 99.3% |
|  |  | 4.00 | 3.91±0.304 | 7.8% | 97.8% |
| Long Stability (Sample placement after 126 days at -80℃) | VK1 | 0.15 | 0.138±0.0216 | 15.7% | 91.9% |
|  |  | 4.00 | 4.04±0.388 | 9.6% | 101.1% |
|  | MK4 | 0.15 | 0.15±0.0134 | 9.0% | 99.7% |
|  |  | 4.00 | 3.91±0.27 | 6.9% | 97.8% |
